# Non-Hermitian photonics promises exceptional topology of light

**DOI:** 10.1038/s41467-018-05175-8

**Published:** 2018-07-10

**Authors:** Bikashkali Midya, Han Zhao, Liang Feng

**Affiliations:** 1 Department of Materials Science and Engineering, University of Pennsylvania, Philadelphia, PA 19104 USA; 2 Department of Electrical and Systems Engineering, University of Pennsylvania, Philadelphia, PA 19104 USA

## Abstract

The band degeneracy, either the exceptional point of a non-Hermitian system or the Dirac point associated with a topological system, can feature distinct symmetry and topology. Their synergy will further produce more exotic topological effects in synthetic matter.

## Introduction

Symmetry and topology are fundamental notions existing in all kinds of natural systems, from spiral galaxies and hurricanes to amino acids and molecules and non-trivial topologically protected electronic states in condensed matter. For example, a stream of linearly polarized photons is typically trivial in its symmetry. But its full-vector nature gives light the full capability of creating and carrying unique symmetry and topology, which allows it to interact with the symmetry and topology of a physical system. Consequently, engraving intriguing symmetry and topology with exotic light states is becoming more and more popular for optics and photonics research. Today, many studies of artificial photonic materials are primarily based on two concepts taken from quantum physics: topological photonics where unique structural topology allows edge/interface states in Hermitian systems^1^ to propagate without scattering; and non-Hermitian photonics with exotic effects based on a range of quantum symmetry paradigms like parity-time symmetry^2^. The fundamental basis behind both of these arises from the mathematical equivalence between the Schrödinger equation in quantum mechanics and the wave equation in optics. As a consequence, both areas can be related to the geometrical nature of spectral degeneracies.

### Exceptional points in non-Hermitian systems

When the Hamiltonian of a conservative system (i.e. a Hermitian system) depends on parameters, the adiabatic theorem states that the system can return to its initial position, apart from a global phase change known as Berry phase, after a slow cyclic evolution in parameter space. The phase factor is dictated entirely by the geometric property of the encircling loop, which reflects the topology of the system.

**Fig. 1:**
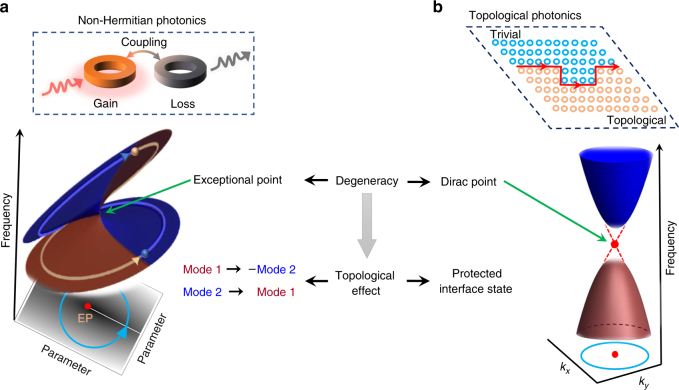
The topology of exceptional point and Dirac point degeneracies. A generic photonic non-Hermitian system is composed of coupled gain and loss materials such as ring resonators (inset of **a**). An exceptional point (EP) can appear in the frequency spectrum of such a system at some critical values of the coupling and the gain/loss coefficient. The topological properties of a second-order EP are best understood by two Riemann surfaces (shown in blue and brown colours) connected at the square root branch point. The two surfaces represent two eigenfrequencies with complex values. An encircling of the EP can be induced by suitable parameter changes. Starting on the upper surface, one ends up on the lower surface after one round and vice versa (the states move smoothly from one surface to the other). Encircling twice brings one back to the initial point, but the modes acquire a π-Berry phase; after 4 cycles, the modes return to their starting phases. For a non-adiabatic encircling, the states suddenly jump from one surface to the other, leading to different final states depending on the direction of encircling (not shown). A schematic of a topological band structure near the Dirac point degeneracy is shown in (**b**). The Berry phase, calculated over a closed loop in the Brillion zone, is related to the topological invariant, known as the Chern number. The gapless edge mode (red dashed line) connects the conduction and valence band. Such an edge state propagates along the interfaces formed by topologically different materials (see red line in the inset). Between the panels, we schematically point out the fundamental degeneracies associated with non-Hermitian and topological photonics

However, counterintuitive effects, appear in certain energy non-conserving systems —systems with open boundaries, energy gain (e.g. by laser pumping) and loss (material absorption or light radiation)—which have an exceptional point (EP) degeneracy in their complex spectra. An EP occurs when the system parameters are tuned to a critical point at which two or multiple resonance frequencies, their line-widths and associated mode profiles coalesce simultaneously^3^. A single encircling of the second-order EP (where exactly two frequencies coalesce), under a stationary condition, results in swapping of the eigenstates, but only one of them acquires a π-Berry phase shift. This peculiarity is a direct outcome of the topology associated with the branch-point character of the complex eigenfrequencies (Fig. 1a). The emergence of the π-phase shift, as explained later, has a unique consequence: it offers a topological fractional charge, something that only occurs in non-Hermitian systems. More remarkably, a dynamical encircling of an EP leads to a robust asymmetric jump to a final state that is different for a clockwise and a counterclockwise loop. The resulting asymmetric transmission is a result of the breakdown of the adiabaticity. Topological operations close to an EP—that is, performed in a closed circuit irrespective of its precise geometry—thus achieve certain goals depending solely on whether the encircling is adiabatic or non-adiabatic in nature. This topological energy transport around an EP has been experimentally verified in a microwave cavity experiment^4^, a designed waveguide structure^5^ and an optomechanical setup^6^ (among others) and may find applications in quantum control and switching.

### Dirac points in topological photonics

Hermitian photonics crystals or waveguide arrays can be engineered to possess a different type of degeneracy: a Dirac point, that is, a linear band crossing point in the Brillouin zone where the conduction and valence bands touch each other (Fig. 1b). The Dirac point can separate distinct topological phases and mark the creation or annihilation of gapless edge modes^1,7^, but trivial Dirac points without a topological transition also exist.

In a topological insulator, gapless edge modes are protected by time-reversal and crystal symmetry, and the band structure of a topological insulator is topologically distinct from that of a conventional insulator. The Hamiltonians describing this band structures cannot be deformed smoothly into each other without closing the energy gap at the degeneracy, in the same topological manner as a Möbius strip cannot be deformed into a hollow cylinder without cutting the surface. The topological phases can be characterized by the Chern number *C*, which is a topological quantum number. It is defined by the Berry phase equal to 2π*C*, which can be calculated by considering the integral of the Berry curvature around a band degeneracy over a closed manifold in the Brillion zone. Because the Chern number is insensitive to the local geometry of the manifold, states at an interface of two topologically distinct materials (characterized by different Chern numbers), can propagate undisturbed, even in the presence of disorder.

Despite the striking similarities between non-Hermitian and topological photonics, for instance—both have degeneracies and non-vanishing Berry phase—the two have a few characteristics that set them apart: firstly, the modes become parallel at an EP, whereas they remain linearly orthogonal at a Dirac point; secondly, the Dirac point manifests robust interface states—a topological consequence very different from the adiabatic state flipping at an EP. Nevertheless, the similarity between the two types of degeneracies has recently been revealed by a complex perturbation, in terms of radiation loss, to an otherwise conservative photonic crystal slab. The perturbation forces the Dirac point to bifurcate in a continuous ring of EPs, forming an exceptional ring in reciprocal space^8^.

### Combining non-Hermitian and topological photonics

The dichotomy and similarity between topological characters of a non-Hermitian EP and a Hermitian band degeneracy leads to a natural question: what benefits can be harvested from the interplay between optical non-Hermiticity and topological physics? A straightforward answer is that the efficient manipulation of gain and loss using non-Hermiticity can selectively enhance the desired topological interface state^9^, making it stand out from other topologically trivial bulk states, even in a structure of the same topological order^10^. Another example is the recent experimental demonstration of topological insulator lasers^11^, which is energy-efficient because of its non-Hermiticity, and at the same time its topological nature makes it robust against defects.

**Fig. 2:**
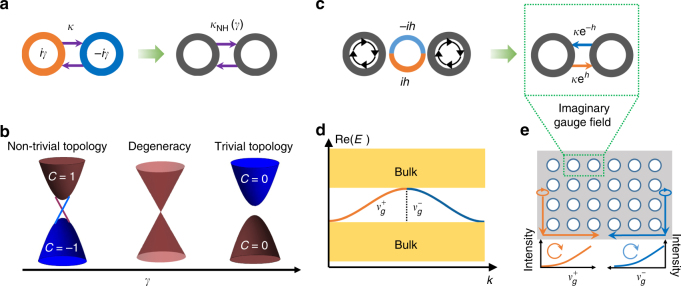
Novel functionalities from the interplay between optical non-Hermiticity and topological photonics. **a** shows the unit cell of a coupled resonator array, with hopping rate *κ* and onsite gain/loss coefficient *γ*, which is equivalent to its Hermitian counterpart with a modified coupling $\kappa _{{\mathrm{NH}}} = \sqrt {\kappa ^2 - \gamma ^2}$. The non-Hermiticity controlled coupling accomplishes the topological phase transition from the trivial to non-trivial band structures via the degeneracy by tuning the gain/loss coefficient as shown in (**b**). Here *C* represents the Chern number related to the bulk band. **c** shows a typical unit cell of a coupled resonator array subjected to a pseudo-spin-dependent imaginary gauge field engineered by an anti-resonance link ring with gain on the lower half and loss on the upper half. This coupling scheme is equivalent to two site resonators with asymmetric hopping rates: the clockwise circulating light hops from left to right with a rate of exp(*h*), while it hops from right to left with a much smaller rate of exp(−*h*), where *h* is the single-pass amplification/attenuation of the lower/upper halves of the link ring. **d** shows the topological pseudo-spin subbands, subjected to the imaginary gauge field, containing the amplifying propagation state with forward group velocity $v_g^ +$ (orange curve) and the attenuating propagation state with backward group velocity $v_g^ -$ (blue curve). Owing to the imaginary gauge field, only the forward propagation can be observed in real space while the backscattering is absorbed in the link rings, resulting in robust one-way propagation, as shown in (**e**)

Beyond the non-Hermitian topological coupling in mode selection, non-Hermitian photonics promises an extra flavour to expand the parameter space of topological photonic structures into the full complex domain, paving the way towards unexplored mechanisms of bulk-edge correspondence in open optical systems^12,13^. To conceptually visualize the prospect, we have shown in Fig. 2a and b a scheme to induce a topological phase transition solely by non-Hermitian engineering, where the onsite gain and loss effectively transform into non-Hermiticity-controlled coupling. This new degree of freedom enables the couplings to be tuned, which is useful for lifting the degeneracy and results in topological states from the band inversion. For example, when the non-Hermitian coupling between the two elements of the dimers continuously increased by reducing the magnitude of the gain/loss coefficient, the trivially gapped bands reach the degeneracy and, upon further reduction, open a topological non-trivial gap. This convenient manipulation of gain/loss via pump-controlled or gate-controlled optical absorption helps reconfigure the topological order that is otherwise as good as unchangeable in the Hermitian limit. The intimate interplay between non-Hermitian physics and topological photonics therefore offers novel solutions to control the path and number of topological states, promising robust switches and routers for next-generation optical communications.

Additionally, non-Hermiticity can be incorporated to attain unique topological features that have no counterpart in Hermitian systems. For example, the π-Berry phase associated with the EP in non-Hermitian systems has been shown to possess a half-integer topological charge^14^. This may open the door to versatile generation of half-twisted vector beams. Furthermore, the conceptual hybridization of topology and non-Hermiticity promises a new class of topological phase transitions: when two oppositely charged EPs merge, the topological charge can disappear without opening a gap^12^. Moreover, in contrast to the synthetic (real) gauge field with a hopping phase imbalance in Hermitian configurations, strategic non-Hermitian engineering can produce an imaginary gauge field^15^. Photons subject to such an imaginary vector potential accumulate an imaginary phase along their trajectory, becoming amplified or attenuated depending on the direction in which they travel. As shown in Fig. 2c–e, such an imaginary gauge field and directional coupling can be implemented by linking two resonators with a non-Hermitian-engineered anti-resonance ring. For sufficiently large values of the gauge field perfect transmission, even in the presence of disorder, can be achieved because back-scattered waves are evanescent (rather than propagating) when an imaginary gauge field is applied. An intriguing feature of the imaginary gauge field is its ability to transform a protected interface state into an extended state, thus preventing localization and restoring mobility.

Despite the demonstrated transformative potential for fundamental and applied photonics, research efforts on the synergy between quantum symmetries and topology are still in their infancy. One largely unexplored area is the consideration of symmetry paradigms other than non-Hermiticity. For example, supersymmetry, which regulates the relation between fermions and bosons in quantum field theory, is proven to be a faithful tool for spectral engineering of certain physical systems. In general, symmetry engineering of photonics offers a powerful toolbox for topological photonics research by providing a platform to design materials with topological properties on demand.
